# Evaluation of the protective effects of icariin on nicotine-induced reproductive toxicity in male mouse –a pilot study

**DOI:** 10.1186/s12958-020-00620-0

**Published:** 2020-06-18

**Authors:** Guochao Ni, Xuhui Zhang, Seth Yaw Afedo, Rong Rui

**Affiliations:** 1 Department of Clinical Veterinary Medicine, College of Veterinary Medicine, Nanjing Agricultural University, Nanjing, 210095 Jiangsu China; 2 Co-Innovation Center for Sustainable Forestry in Southern China, College of Forestry, Nanjing Forestry University, Nanjing, 210037 Jiangsu China

**Keywords:** Icariin, Nicotine, Sperm density, Testosterone, Antioxidant enzyme, Male mice

## Abstract

**Background:**

Nicotine, a pharmacologically active component of tobacco adversely affects the male reproductive system and fertility whereas icariin (ICA), the main active ingredient in *Epimedium herba* has been used in the treatment of several male reproductive problems. This study aimed at evaluating the protective or ameliorative effect of ICA against reproductive toxicity induced by intraperitoneal injection of nicotine in mice.

**Methods:**

Using simple random allocation, forty male mice were randomly divided into 4 groups: control (received 0.35 mL physiological saline via gastric gavage), nicotine (0.75 mg/kg BW/day intraperitoneally), ICA (75 mg/kg BW/day gastric gavage), and nicotine plus ICA (nicotine, 0.75 mg/kg BW/day intraperitoneally + ICA, 75 mg/kg BW/day gastric gavage) group. After 35 days of treatment, the mice were weighed, sacrificed, and their reproductive organs (testis and epididymis) were collected and examined for further studies.

**Results:**

The nicotine-treated group showed significantly decreased epididymal sperm density and serum testosterone concentration relative to the control group. Nicotine also caused oxidative damage shown by significant reduction in the activities of antioxidant enzymes and elevation in Malondialdehyde (MDA) levels. ICA on the other hand, improved the reduction in sperm density, hormone levels, and activities of antioxidant enzymes altered in the nicotine treated mice.

**Conclusions:**

These findings indicate that nicotine-induced reproductive toxicity and oxidative damage on male reproductive tissues could be attenuated by ICA.

## Introduction

Tobacco contains numerous compounds among which nicotine is the most addictive and toxic component. Among users of tobacco, nicotine plays a significant role in the development of cardiovascular disorders, pulmonary diseases, lung cancer, and many other diseases [1, 2]. Nicotine has also been known to cause oxidative stress (OS) by inducing the generation of reactive oxygen species (ROS) in tissues [3]. In addition, it adversely affects the male reproductive system and fertility [4]. Several studies have also reported that nicotine adversely affects spermatogenesis, epididymal sperm count, motility, and the fertilizing potential of sperms [5–8]. Nicotine causes disturbances in the function of Leydig cells, thus diminishing the production of testosterone [8].

Medicinal plants and their active ingredients have received greater attention as potential anti-peroxidative agents. Icariin (ICA), (C_33_H_40_O_15_; molecular weight: 676.67), the main active flavonoid glucoside from *Epimedium herba* is associated with a wide range of pharmacological and biological activities, including anti-inflammatory, antidepressant, anti-tumor activity, antioxidant effect, estrogenic activity, cardiovascular protection, enhancement of bone healing and neuroprotection, immunoregulation, and improved sexual function [9–12]. Experiment on animals has revealed that ICA improves erectile function when administered to aged male rats [13]. ICA supplementation could also elevate exercise endurance as it provides protective effects on exercise-induced OS [14, 15]. The testes contain an elaborate array of antioxidant enzymes and free radical scavengers which ensure that its twin spermatogenic and steroidogenic functions are not impacted by OS [16, 17].

ICA has testosterone mimetic properties. Testosterone plays a leading role in both morphological development and reproductive function in the testis [18]. There are several separate reports on nicotine (as a cause of fertility problems in males) and ICA (as a factor in enhancing male reproduction and fertility) [1, 4, 13, 19, 20], but there are no reports on the effects of ICA on nicotine-induced reproductive toxicity. Therefore, this present study was conducted to evaluate the possible protective effect of ICA against nicotine-mediated reproductive toxicity and OS in mice through assessment of reproductive function and activities of the main antioxidant enzymes.

## Materials and methods

### Drugs and chemicals

Nicotine ditartrate was purchased from Adooq Bioscience Co., Ltd. (Irvine,CA, USA) and ICA from Ze Lang Co., Ltd. (Nanjing, China). The SYBR® PrimeScript® real time-polymerase chain reaction (RT-PCR) Kit (Perfect Real Time) was purchased from TaKaRa Biotech (Liaoning, China). Superoxide dismutase (SOD), glutathione peroxidase (GPx), and malondialdehyde (MDA) assay kits were obtained from Jiancheng Bioengineering Institute (Nanjing, China), and testosterone (T) Radioimmunoassay (RIA) Kit from Abcam (Cambridge, UK).

### Ethical approval

All procedures and protocols involving animals were in accordance with the Animal Ethics Procedure and Guidelines of the People’s Republic of China and the Guide for the Care and Use of Laboratory Animals. All animal procedures were also approved by the Institutional Animal Care and Use Committee (IACUC) of Nanjing Agricultural University with Permit No. 2018CB114306.

### Animals and experimental design

Forty healthy male Kunming mice (8-weeks-old) were purchased from Beijing Vital River Laboratory Animal Technology Co., Ltd. (permit number SCXK-Jing 2016–0011). The mice were purchased 1 week prior to the study, and for the purposes of acclimatization, all mice were routinely raised in a clean area with normal room temperature and fed with standard mouse feed and ordinary water ad libitum. A sample size calculation was made according to Daniel (1999) [21]. The minimum sample was calculated based on the formula: n = Z^2^ P (1-P) / d^2^, where n = sample size, Z = Z -score, P = margin of error, and d = standard deviation. The Z-score is a constant value automatically set based on the decided confidence level. Accordingly, the necessary sample from an unknown population was determined as 385 (95% confidence level, 50% standard deviation, and a 5% margin of error). However, it was not possible to treat or handle tissue samples from 385 mice in 1 day. Therefore, we conducted a pilot study with 40 mice divided into 4 treatment groups as previously suggested by Zhou et al. (2006) [22].

A simple random allocation was used to assign 10 mice to each group. Briefly, each mouse had equal chance to be selected as part of any of the groups to avoid statistical bias. All mice were marked on the ear; from 1 to 40 and paper cards bearing marks of numbers from 1 to 40 were also designed. Using a random number table, the mice were randomly picked and assigned (allocated) to any of the 4 groups under consideration until each group had a total of 10 mice. Group I served as the control, mice assigned to this group were administered 0.35 mL physiological saline via gastric gavage. Group II mice received intraperitoneal injection of nicotine tartrate (dissolved in 0.9% physiological saline) at 0.75 mg/kg BW/day. The mice assigned to group III were given ICA at 75 mg/kg BW/day through gastric gavage, and finally, group IV mice received intraperitoneal injection of nicotine tartrate (dissolved in 0.9% physiological saline) at 0.75 mg/kg BW/day and then administered ICA at 75 mg/kg BW/day via gastric gavage.

Each mouse was administered once a day for 35 consecutive days. The dosage of nicotine corresponded to the daily nicotine intake in people who smoke 10–20 cigarette sticks per day [8, 23]. The mice were weighed weekly to adjust the gavage or injection volume.

### Dissection of testes and epididymides

At the end of treatment, the mice were sacrificed by cervical dislocation. The testes and epididymides were quickly collected and weighed. Testes samples for histopathological examination were kept in Bouin’s fixative to preserve normal morphology and facilitate further processing into paraffin blocks. Testes samples collected for assays of biochemical analysis and real-time PCR were snap-frozen in liquid nitrogen and stored at − 80 °C until they were used for further experiments. The epididymides were used for sperm density analysis. The organ (testicular / epididymal) index was determined as follows:

$$ \mathrm{Organ}\ \left(\mathrm{testicular}/\mathrm{epididymal}\right)\ \mathrm{index}=\mathrm{organ}\ \mathrm{weight}/\mathrm{body}\ \mathrm{weight}\times \mathrm{1,000}. $$

### Epididymal sperm density

The left epididymis was used for sperm counting. The epididymides were cut into small pieces after which epididymal spermatozoa were collected and immersed in 4 mL of human tubal fluid (HTF) in a petri dish [24, 25]. This was incubated in 5% CO_2_ for 15 min at 37 °C, and homogenization-resistant sperms were counted using haemocytometer. The number of cells in at least 2 of the large corner squares (1 mm^2^) was counted [26].

### Histopathological examination

The right testis was fixed in Bouin’s solution for 24 h and processed by standard histological procedures. Briefly, specimens were dehydrated in xylene and embedded in paraffin wax, 5 μm thick slices were cut from each sample onto a glass slide, and stained with haematoxylin-eosin (HE) routinely. Testicular morphology and structure were observed under the microscope.

### Measurement of serum testosterone

When treatment ended, mice were placed in a plastic air-tight container, together with ketamine (100 mg/kg) and xylazine (10 mg/kg) soaked tissue paper and covered immediately for deep anesthesia, after which blood samples were obtained by cardiac puncture. The collected blood samples were centrifuged at 2000×g for 10 min to separate the serum. The serum was stored at − 20 °C until they were used for further analysis. The serum testosterone concentrations were measured using a radioimmunoassay (RIA) kits.

### Determination of SOD, GPx activity, and MDA level

The testicular tissues stored at − 80 °C were homogenized after adding pre-cooled 0.9% physiological saline in the ratio of 1:9. When testicular tissues were disrupted, the homogenate was centrifuged at 5000 rpm for 10 min at 4 °C. The supernatant was used for assays involving superoxide dismutase (SOD), glutathione peroxidase (GPx), and MDA according to manufacturer’s instructions.

### Quantitative real-time PCR analysis

The expression levels of 3β-hydroxysteroid dehydrogenase (3β-HSD), 17β-hydroxysteroid dehydrogenase (17β-HSD), steroidogenic acute regulatory protein (StAR), cytoplasmic superoxide dismutase 1 (SOD1), mitochondrial superoxide dismutase 2 (SOD2), extracellular superoxide dismutase 3 (SOD3), and glutathione peroxidase 1 (GPx1) mRNA in testicular tissues were assessed by qRT-PCR. Primers (Table 1) were synthesized at Shanghai Invitrogen Biotech Co. Ltd. Total RNA was extracted from testicular tissues using Trizol reagent (Invitrogen, CA, USA), and according to manufacturer’s instructions. The quality and quantity of the RNA preps were determined by gel electrophoresis and Nanodrop ND-1000 spectrophotometer (Nanodrop Technologies Inc., DE, USA). Each real-time PCR reaction was carried out in triplicate in a 20-μL reaction mixture (2 μL cDNA, 6.8 μL H_2_O, 10 μL SYBR® Premix Ex Taq™, and 0.6 μL of each 10 μM forward and reverse primers). The PCR program was 30 s at 95 °C followed by 40 cycles of 5 s at 95 °C, 31 s at 60 °C. A melting curve was generated at the end of every run to ensure product uniformity (95 °C for 15 s, 60 °C for 15 s, 95 °C for 15 s). The relative expression of target genes was calculated using 2^−ΔΔCt^ method with β-actin as an internal control.

**Table 1:** Primer sequences used in real-time polymerase chain reaction (RT-PCR)

Genes	Forward primer	Reverse primer	Lengths of amplicons (bp)
β-actin	TACTGAGCTGCGTTTTACACC	TCCTGAGTCAAAAGCGCCAA	177
3β-HSD	AGATAATCCTGAATGGCAACGA	TTTGCCCGTACAACCGAGA	245
17β-HSD	TGGGTGCTGTGTTGGATGTG	TGGCACAGTACACTTCGTGG	153
StAR	TGCGCTTAAATCTCCTAGCTC	TGGCTATCCTTCTGTGTAGACC	195
SOD1	AAGCGGTGAACCAGTTGTGT	CCGGGCCACCATGTTTCTTA	152
SOD2	AGGAGAGTTGCTGGAGGCTA	TAGTAAGCGTGCTCCCACAC	228
SOD3	GAGAAGATAGGCGACACGCA	GAGAACCAAGCCGGTGATCT	156
GPx1	ACAGTCCACCGTGTATGCC	CGTTCATCTCGGTGTAGTCCC	146

β-actin, Beta-actin; 3β-HSD, 3Beta-Hydroxysteroid dehydrogenase; 17β-HSD, 17Beta-Hydroxysteroid dehydrogenase; StAR, steroidogenic acute regulatory protein; SOD1, cytoplasmic superoxide dismutase 1; SOD2, mitochondrial superoxide dismutase 2; SOD3, extracellular superoxide dismutase 3; GPx1, Glutathione peroxidase 1

### Statistical analysis

The results were expressed as mean ± standard deviation (SD) of the mean and statistically analyzed by one-way analysis of variance (ANOVA) using SPSS Statistics Package 25 (Chicago, IL, USA) and differences between experimental groups were considered significant at *P* < 0.05.

## Results

### Effects of icariin and nicotine on body weight, testicular, and epididymal weight

Comparisons of the body weights, testicular and epididymal weights, and testicular and epididymal indices between the four study groups of mice are shown in Table 2.

**Table 2:** Effects of icariin and nicotine on body weight, reproductive organ weight and index in the experimental mice

Groups	Initial Body Weight(g)	Final Body Weight(g)	Testicular weight(g)	Testicular index (mg/g)	Epididymal weight(g)	Epididymal index (mg/g)
Control	45.15 ± 2.20^a^	52.04 ± 4.67^a^	0.280 ± 0.023^a^	5.39 ± 0.30^a^	0.106 ± 0.008^a^	2.05 ± 0.11^a^
Nicotine	46.03 ± 2.27^a^	53.49 ± 4.10^a^	0.279 ± 0.019^a^	5.22 ± 0.15^a^	0.107 ± 0.011^a^	2.00 ± 0.14^a^
ICA	44.60 ± 3.35^a^	50.68 ± 3.76^a^	0.302 ± 0.023^b^	5.97 ± 0.39^b^	0.104 ± 0.007^a^	2.05 ± 0.13^a^
ICA + Nicotine	44.13 ± 2.56^a^	52.09 ± 5.01^a^	0.288 ± 0.021^ab^	5.56 ± 0.45^ab^	0.105 ± 0.008^a^	2.02 ± 0.11^a^
*P*-value	0.43	0.57	0.07	0.00	0.87	0.71

Values are expressed as means ± S.D.; (n = 10). Values not sharing a common superscript differ significantly at *P* < 0.05. ICA, Icariin

### Effects of icariin and nicotine on testicle morphology

**Fig. 1:**
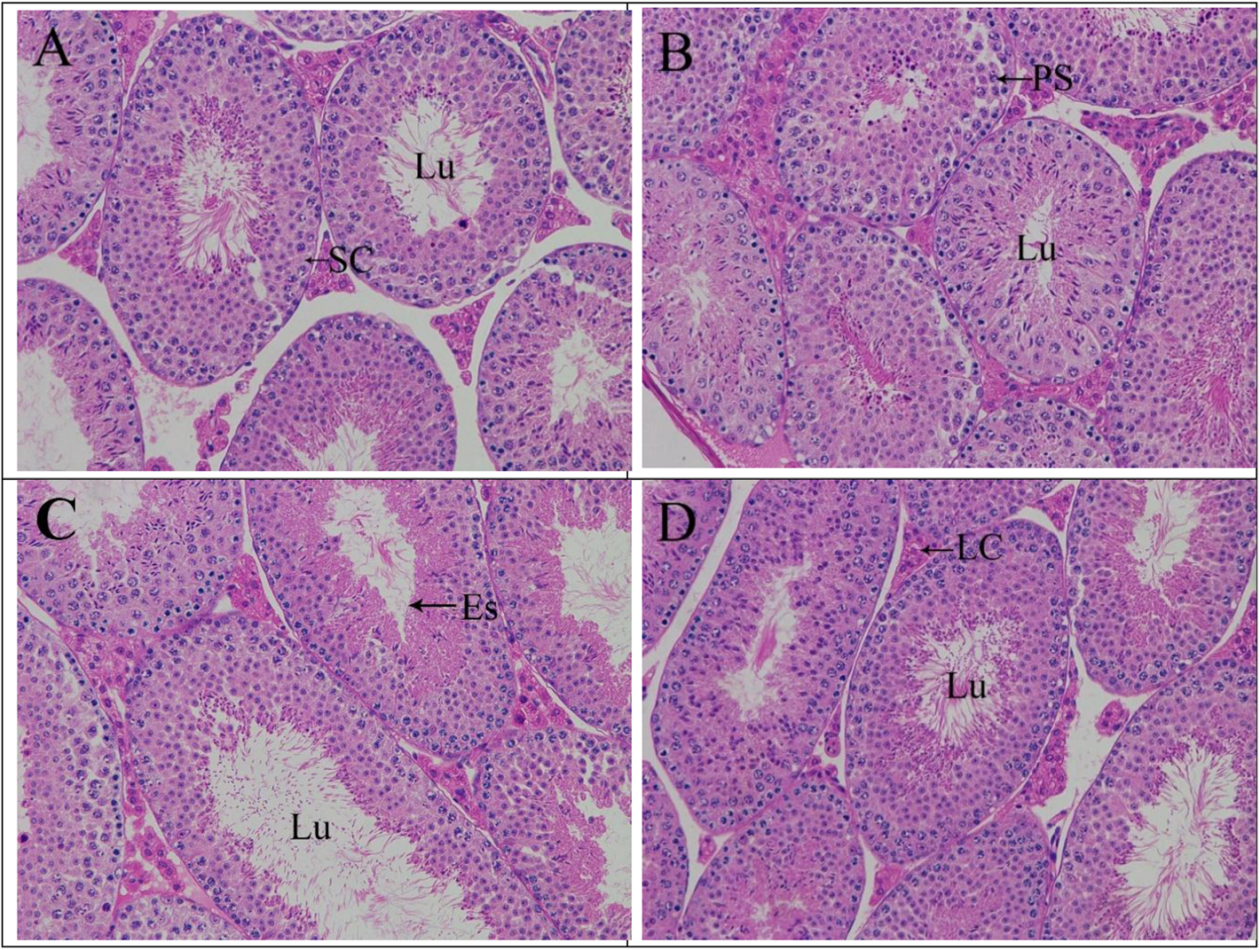
Haematoxylin and eosin stained sections of testes in different groups (× 200). **a** Control, (**b**) 0.75 mg/kg BW Nicotine, (**c**) 75 mg/kg BW Icariin, (**d**) 0.75 mg/kg BW Nicotine plus 75 mg/kg BW Icariin. Lu, lumen; Sc, Sertoli cells; Lc, Leydig cells; PS, primary spermatocyte; ES, early spermatid; BW, body weight

Figure 1 shows representative photomicrograph of the testes from the different groups. The seminiferous tubule basement membrane was continuous and complete, and the germ cells had normal morphology and regular arrangement in all groups (Fig. 1a, b, c, and d). Primary spermatocytes and early spermatids were also observed in the control, ICA, and nicotine plus ICA treated groups. However, developed lumina in the seminiferous tubules from the control (Fig. 1a) and ICA (Fig. 1c) treated mice showed complete spermatogenesis and high sperm density compared with that in the nicotine treated mice (Fig. 1b) where low sperm density was observed in constricted lumina. Upon co-treatment with ICA, the lumina of the seminiferous tubules showed complete spermatogenesis and improved sperm density (Fig. 1d).

### Effects of icariin and nicotine on epididymal sperm density,serum testosterone concentration, MDA levels, activities of SOD, and GPx in mice testes

Comparisons of epididymal sperm densities, serum testosterone levels, and testicular tissue OS indices between the four study groups of mice are shown in Table 3.

**Table 3:** Testicular tissue SOD, GPx activities and MDA level, epididymal sperm density, and serum testosterone levels

Groups	SOD (U/mg protein)	GPx (U/mg protein)	MDA (nmol/g protein)	Epididymal sperm density (10^7^/mL)	Testosterone (ng/mL)
Control	159.35 ± 8.15^a^	23.26 ± 2.72^a^	3.97 ± 0.45^a^	1.02 ± 0.09^a^	0.341 ± 0.057^a^
Nicotine	147.34 ± 7.38^b^	22.83 ± 1.46^ac^	4.63 ± 0.67^b^	0.83 ± 0.07^b^	0.273 ± 0.035^b^
ICA	175.79 ± 9.03^c^	26.74 ± 1.95^b^	3.23 ± 0.61^c^	1.55 ± 0.31^c^	0.675 ± 0.105^c^
ICA+ Nicotine	169.55 ± 4.19^c^	24.13 ± 3.12^ab^	3.82 ± 0.57^a^	1.25 ± 0.19^c^	0.494 ± 0.065^d^
*P*-value	0.00	0.01	0.00	0.00	0.00

*SOD* superoxide dismutase; *GPx* glutathione peroxidase; *MDA* malondialdehyde; *ICA* Icariin. All data was expressed in mean ± SD; (n = 10); Values not sharing a common superscript differ significantly at *P* < 0.05

### Effects of icariin and nicotine on mRNA expression levels of steroidogenic and antioxidant genes in mice testis

**Fig. 2:**
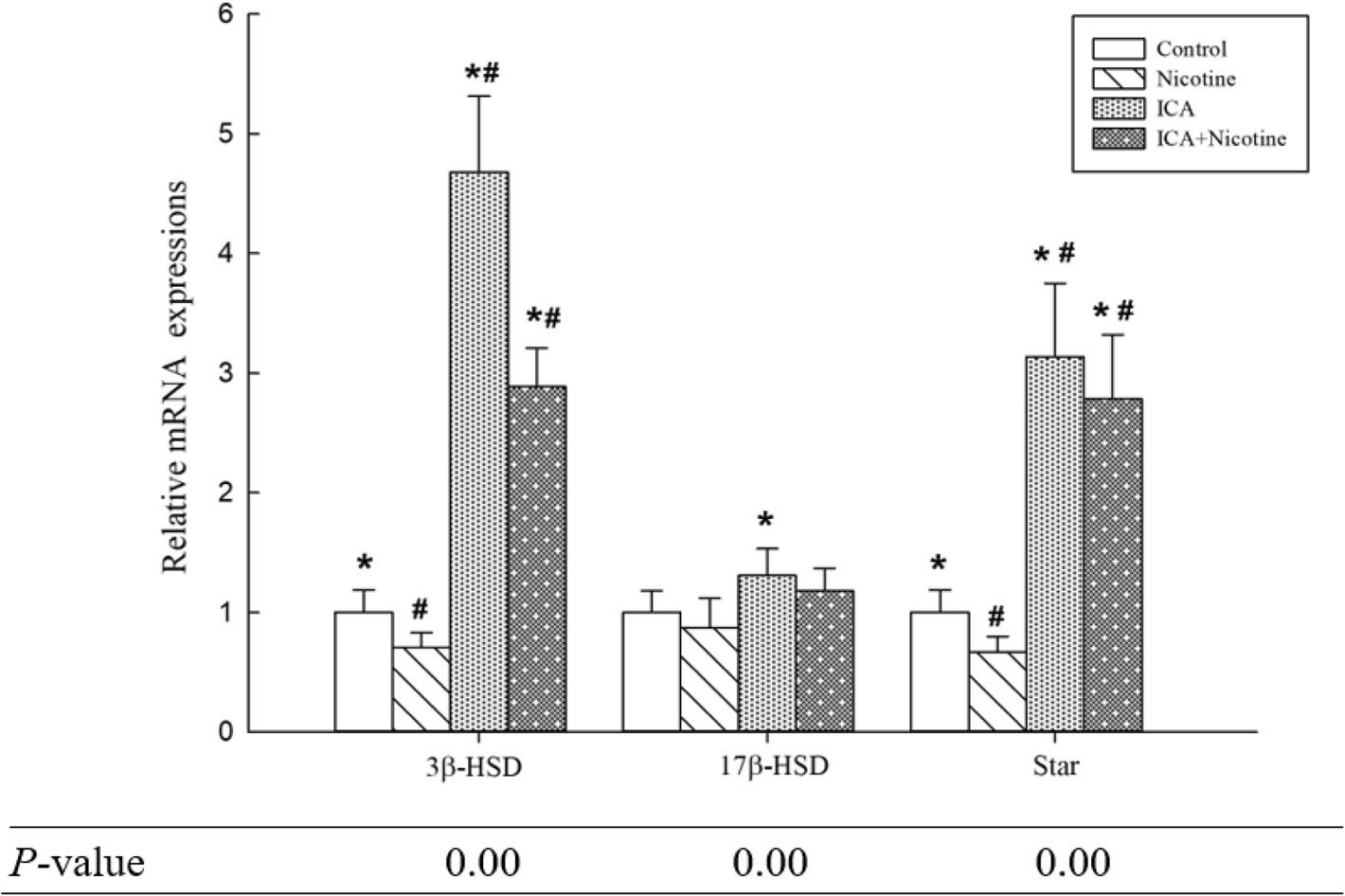
Expressions of 3β-HSD, 17β-HSD, and StAR mRNA in the testes of control, nicotine, icariin, and nicotine plus icariin groups. The mRNA levels were normalized using β-actin mRNA as internal control. The mRNA levels of the control group were set as 1. #*P* < 0.05 compared with the control group, *P < 0.05 compared to the nicotine-treated group. (*n* = 10). 3β-HSD, 3β-Hydroxysteroid dehydrogenase; 17β-HSD, 17β-Hydroxysteroid dehydrogenase; StAR, steroidogenic acute regulatory protein

**Fig. 3:**
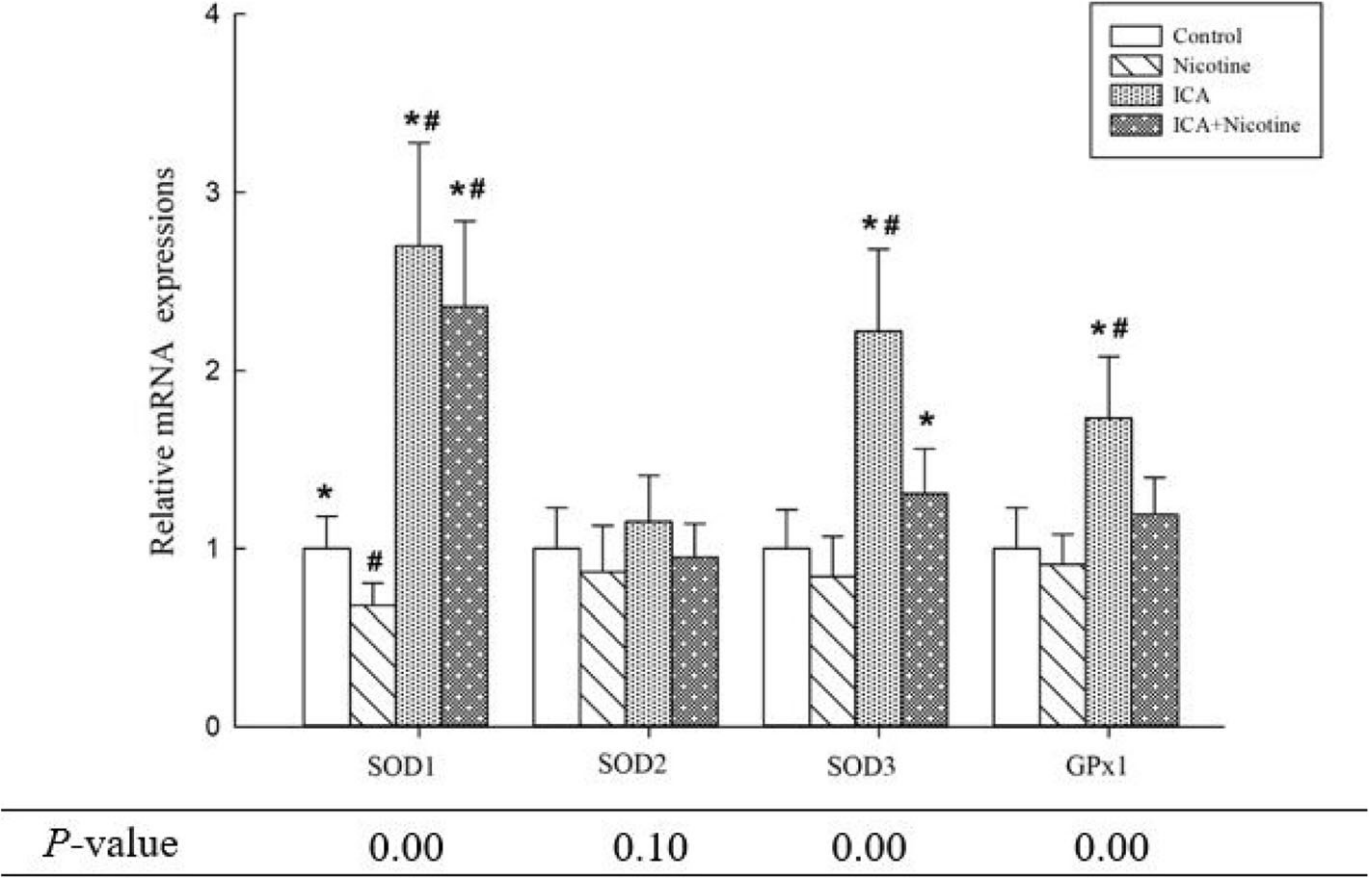
Expressions of SOD1, SOD2, SOD3, and GPx1 mRNA in the testes of control, nicotine, icariin, and nicotine plus icariin groups. The mRNA levels were normalized using β-actin mRNA as internal control. The mRNA levels of the control group were set as 1. #*P* < 0.05 compared to the control group, *P < 0.05 compared to the nicotine-treated group. (n = 10). SOD1, cytoplasmic superoxide dismutase 1; SOD2, mitochondrial superoxide dismutase 2; SOD3, extracellular superoxide dismutase 3; GPx1, Glutathione peroxidase 1

Comparisons of mRNA expression levels of steroidogenic and antioxidant genes in mice testis between the four study groups of mice are shown in Figs. 2 and 3.

## Discussion

This pilot study evaluated the protective effects of ICA on reproductive toxicity induced by intraperitoneal injection of nicotine in adult male mice. Thirty-five consecutive days of nicotine treatment caused reproductive toxicity; as a result of decreased sperm concentration in the epididymis and increased MDA levels in the testis; however, co-treatment with ICA reversed these trends. This is probably because ICA improved testosterone concentration in blood and antioxidant enzyme activities in the testis thereby ameliorating the adverse effect of nicotine.

The result of our study also demonstrated that nicotine and ICA administration had no significant effect on body weight and epididymal weight/index. In 2014, Chen et al. reported that body weight of rats treated with ICA did not change significantly in any group, suggesting ICA has no obvious effects on adult rat growth [13]. Other studies also reported same findings and our results from ICA treatment were in concordant [10, 27]. On the other hand, Seema (2007) and Hasanzadeh (2017) reported that exposure to nicotine decreased body weight significantly in rats and mice respectively [2, 11]. Also, Williamson (1991), had earlier intimated that, in humans, cigarette smoking results in weight loss, and the smokers gained weight, when they quit smoking [28]. Our findings on nicotine were not the same and we speculate that these differences in weight results could be due to differences in dosage of nicotine administered, the administration route, duration of experiment, or the age of experimental animals.

Testosterone is essential for sexual differentiation, maintenance of spermatogenesis, and expression of male secondary sex characteristics [29]. Holdcraft (2004) reported that testosterone plays a leading role in both morphological development and reproductive function in the testis [18]. We found marked decreased serum testosterone concentrations and epididymal sperm density in the nicotine treated group. This finding was also in agreement with previous studies [4, 9, 10, 30]. However, co-treatment with ICA reversed these trends. Previous studies found that administration of *E. brevicornum* Maxim for 15 d dramatically increased testosterone levels compared with that in the control group and serum testosterone concentrations of ICA treated-rats significantly increased with increasing dosage [6, 13]. A report in human studies has revealed that the levels of nicotine detected in serum and semen of smokers showed positive correlation with the dose of smoking exposure [4]. In androgen-producing Leydig cells, nitric oxide/cyclic guanosine mono phosphate (NO/cGMP) signaling pathway participates in the regulation of steroidogenic activity [31]. Phosphodiesterase type 5 inhibitors (PDE5-Is) can inhibit PDE5, an enzyme responsible for the degradation of cGMP. Hence PDE5-Is could be involved in the activation of steroidogenic pathways and testosterone secretion. PDE5 is identified in Leydig cells [32]. Zhang et al. in 2012 described ICA as a PDE5-I with the potential to increase cellular cGMP through enhancement of nitric oxide synthase (NOS) in diabetic erectile dysfunction (ED) rats’corpus cavernosum tissue [33]. Considering the effects of ICA on the production of testosterone we speculate that ICA could play an important role in the activation of NO/cGMP pathway. A future study on the mechanism through which ICA improves testosterone production is therefore worth considering.

Testicular 3β-HSD and 17β-HSD plays a pivotal role in the biosynthesis of testosterone, which is a prerequisite for sperm production and maturation [34]. The mRNA expression level of 3β-HSD decreased following nicotine exposure, but the down-regulation tendency reversed as a result of co-treatment with ICA. We also found decreased levels of 17β -HSD activity due to nicotine exposure, meanwhile Pant (2003) and Pushpalatha (2005) earlier reported that male reproductive potentials decreased with decreasing testicular 17β-HSD activity levels following exposure to several xenobiotics in rats [35, 36]. Nevertheless, upon ICA treatment the results showed significant increase in 17β-HSD mRNA expression when compared with the nicotine treated group. StAR as a key transmembrane transport regulator plays a crucial role in testosterone production and its involvement in the transport of cholesterol across mitochondrial membrane is generally considered a rate-limiting step in steroidogenesis. In our study, steroidogenic gene StAR mRNA expression levels decreased following nicotine exposure, but the down-regulation trend reversed, when the adult mice were co-treated with ICA. The production of testosterone therefore could be affected when the mRNA expression levels of 3β-HSD, 17β-HSD, and StAR are up and down-regulated [13, 37]. This study suggests a possible mechanism through which ICA improves testosterone production.

ROS include hydroxyl radicals (•OH), superoxide anion (•O_2_-), hydrogen peroxide (H_2_O_2_), and nitric oxide (NO). ROS overproduction depletes sperm antioxidant system, which leads to a condition of OS. The OS arises when excess free radicals overwhelm the antioxidant defense of the male reproductive tract thereby damaging cells, tissues, and organs [38, 39]. There exist an elaborate array of antioxidant enzymes and free radical scavengers in the testes, and they ensure that the twin spermatogenic and steroidogenic functions are not impacted by OS [16, 17]. In cells, the SOD enzyme rapidly converts superoxide anion (•O_2_-) to less dangerous hydrogen peroxide (H_2_O_2_) so that GPx can decompose H_2_O_2_ to water [40, 41]. The MDA level is frequently used as key indicative index of tissue OS, which results from free radical damage to membrane components of cells [42]. In our study, administration of nicotine caused decreased activities of SOD and GPx enzymes, while MDA levels in the testes increased, leading to testicular OS. These results are in agreement with previous studies [23, 24]. However, co-treatment with ICA significantly inhibited depletion of SOD enzyme activity (as a result of nicotine exposure) and elevated MDA level in the testes. Testicular OS caused by nicotine was therefore eliminated by co-treatment with ICA. The results of this study suggest a possible protective mechanism of ICA against nicotine-mediated reproductive toxicity.

It is well known that SOD serves as an important antioxidant defense against OS. Three forms of SOD, namely, SOD1, SOD2, and SOD3 are present in mammals. More so, GPx1, the most abundant selenoperoxidase, is a key antioxidant enzyme in various cell types [17, 37]. In our study, the mRNA expression of SOD1 in the testes markedly decreased in the nicotine treated group compared with that in the control group, but when co-treated with ICA, these down-regulation tendencies of SOD1 efficiently reversed. The mRNA expression of SOD3 in the ICA and nicotine plus ICA treated group also increased significantly compared with that in the control and nicotine-treated group. Taniyama in 2003 explained that under physiological conditions, superoxide anions are scavenged by SOD1, SOD2, and SOD3 producing a relatively stable ROS H_2_O_2_ [43]. ICA treatment increased the mRNA expression of GPx1 significantly. Reporting on SOD1, SOD2, SOD3, and GPx1, Yuan et al. (2013) indicated that the mRNA expression of these antioxidant genes in mice testes decreased in the presence of cyclophosphamide (CP) which is a cytotoxic agent [44]. The mRNA expression levels of SOD1 and SOD3 decreased upon nicotine treatment, but co-treatment with ICA reversed these trends, this shows that ICA can protect against cytotoxic agents such as nicotine.

### Study limitations

This pilot study is limited by the low number of mice (sample size) used. A study with much larger sample size will be needed to confirm the protective effect of ICA on nicotine-induced reproductive toxicity in male mouse.

## Conclusions

In conclusion, our results show that nicotine-mediated reproductive toxicity in adult mice was ameliorated through ICA treatment, with characteristic actions such as increase secretion of testosterone, antioxidant capacities, and the suppression of OS. These results suggest that ICA could be used as a potential therapeutic agent in cases of decreased sperm production and OS mediated by cigarette smoking.

## Data Availability

The datasets used and/or analyzed during the current study are available from the corresponding author upon reasonable request.

## Abbreviations

ICA: Icariin
BW: Bodyweight
MDA: Malondialdehyde
ROS: Reactive oxygen species
SOD: Superoxide dismutase
GPx1: Glutathione peroxidase 1
OS: Oxidative stress
IACUC: Institutional Animal Care and Use Committee
PCR: Polymerase chain reaction
HTF: Human tubal fluid
HE: Haematoxylin-eosin
RIA: Radioimmunoassay
HSD: Hydroxysteroid dehydrogenase
StAR: Steroidogenic acute regulatory protein
PDE5-Is: Phosphodiesterase type 5 inhibitors
ED: Erectile dysfunction
NOS: Nitric oxide synthase
NO/cGMP: Nitric oxide / cyclic guanosine mono phosphate
CP: Cyclophosphamide
qRT-PCR: Quantitative real time polymerase chain reaction
SD: Standard deviation
ANOVA: Analysis of variance
SPSS: Statistics Package for Social Sciences
